# The relationship between information carrying words, memory and language skills in school age children with specific language impairment

**DOI:** 10.1371/journal.pone.0180496

**Published:** 2017-07-03

**Authors:** Pauline Frizelle, Jennifer Harte, Kathleen O’Sullivan, Paul Fletcher, Fiona Gibbon

**Affiliations:** 1 Department of Experimental Psychology, Oxford University, Oxford, England; 2 Department of Speech and Hearing Sciences, University College Cork, Cork, Ireland; 3 School of Mathematical Sciences, University College Cork, Cork, Ireland; Kyoto University, JAPAN

## Abstract

The receptive language measure *information-carrying word (ICW) level*, is used extensively by speech and language therapists in the UK and Ireland. Despite this it has never been validated via its relationship to any other relevant measures. This study aims to validate the ICW measure by investigating the relationship between the receptive ICW score of children with specific language impairment (SLI) and their performance on standardized memory and language assessments. Twenty-seven children with SLI, aged between 5;07 and 8;11, completed a sentence comprehension task in which the instructions gradually increased in number of ICWs. The children also completed subtests from The Working Memory Test Battery for children and The Clinical Evaluation of Language Fundamentals– 4. Results showed that there was a significant positive relationship between both language and memory measures and children’s ICW score. While both receptive and expressive language were significant in their contribution to children’s ICW score, the contribution of memory was solely determined by children’s working memory ability. ICW score is in fact a valid measure of the language ability of children with SLI. However therapists should also be cognisant of its strong association with working memory when using this construct in assessment or intervention methods.

## Introduction

The term *information carrying words* (ICWs) became commonly used by speech and language therapists (SLTs) and specialist teachers in the UK and Ireland, with the emergence of the Derbyshire Language Scheme, an intervention programme targeting early language skills [1]. The ICW construct continues to be used widely by SLTs in the UK and Ireland as a measure of children’s understanding of language and as a therapeutic tool, in the management of young children with impaired language. A recent online survey conducted by Morgan, Marshall, Harding and Roulstone [2] (which included 231 SLTs in England) revealed that 98% of therapists surveyed, reported using ICWs with preschool and school aged children with primary speech and language impairments. Despite this fact, a literature review has uncovered no research validating ICW as a measure of language or investigating its relationship with other related skills such as memory. This is somewhat surprising given our primary aim to work in the context of a robust evidence base. SLTs who use ICWs as a clinical measure may reasonably assume that it correlates with memory or other language measures but this assumption has never been investigated. It is difficult to interpret ICW as a level of language ability or implement this construct in therapeutic goals when there is no evidence of its validity, for example via its relationship to any other relevant measures.

### ICWs—An overview

The term ICW refers specifically to the number of words which give the specific information in any given instruction or sentence, i.e., the amount of information in an instruction, which the child is required to remember and act upon. It is therefore reasonable that therapists would assume a relationship between children’s receptive ICW performance, their receptive language abilities and their memory abilities. However, calculating the number of ICWs is not as straightforward as simply counting the words that provide information in the sentence. In order to assess the number of ICWs in each instruction the SLT must ascertain how much of the instruction is made redundant by the contextual information available to the child, i.e., given the context, only the words that a child must understand in order to respond correctly are considered to be the ICWs. For example, if a child is offered a plate of biscuits, with the accompanying question ‘Would you like a biscuit?’ it is likely that the child will take one, but the extent to which the child fully understood the language in the question remains unknown. It could be that the child’s response was to the speaker’s tone of voice, their gesture, eye pointing and the overall physical context. In this situation the instruction would be categorized as having no ICWs. If however the child was offered a plate on which there were biscuits, cakes and sweets, with the instruction ‘Take a biscuit’ then we might at least assume that the child understood the name of the food item. The verb *take* might be considered somewhat redundant as it could be interpreted from the context. The number of words that the child must understand in this instruction is therefore categorized as one—the instruction is at a one or single ICW level. Previous instructions given will also affect how the contextual information is interpreted. For example, if a child had already carried out the instruction ‘Give the biscuit to teddy’ and was then told to ‘Take a biscuit’ the word *take* would no longer be considered contextually redundant and the sentence ‘Take a biscuit’ would be considered to have two ICWs. Therefore, depending on the context, the same instruction could be considered to have a different number of ICWs.

It is also important to acknowledge that number of ICWs is an approximate measure within any sentence framework, as the level of vocabulary, the overall length of the instruction, the position of the information within the sentence, the syntactic form and the nature of the context will all affect its level of difficulty. All of these factors can reasonably be assumed to relate to the child’s general language ability and accordingly we would expect a relationship between receptive language abilities and ICW score. It is often assumed in the literature that children’s abilities on one receptive language measure are reflective of their abilities on another. Researchers that language-match two groups of children based on the results from one language assessment are operating on this premise. For example, Donlan, Bishop and Hitch [3] and Montgomery [4] language-matched (receptively) using the test results from the Test for the Reception of Grammar (TROG) [5], Norbury, Bishop and Briscoe [6] using those from the British Picture Vocabulary Scales (BPVS) [7] and Durkin and Conti-Ramsden [8] using the word classes subtest from the Clinical Evaluation of Language Fundamentals (CELF—R) [9], stating that it is used widely in the literature and considered a good indicator of language skills. Other studies have language matched using the expressive measure, mean length of utterance (MLU) (see [10–11]). Research identifying sentence recall as a discriminating marker of specific language impairment (SLI), (sensitive to individual differences in language ability) [12] also suggests that this single measure of language ability is reflective of a more general language competence. Therefore we might predict that ICW score, as an individual measure, will be strongly associated with global language ability. However, this has never been investigated.

It is also noteworthy that when using the ICW construct it is not intended to be a reflection of children’s receptive vocabulary. In fact therapists often ensure that children understand the vocabulary items in the instruction in order that the measure relates to number of ICWs recalled. In this regard it is measuring something quite different from the range of language skills assessed in standardized language measures and we might expect that it would be more closely associated with measures of memory. Many studies have focused on memory in relation to different aspects of receptive language; in standardized tests [13–14], at single word level [15], at sentence level (both simple and complex) [4, 6, 16–20] and in narrative [21]. However there are no studies exploring memory in relation to the ICW construct. An understanding of this relationship would provide us with better knowledge to inform diagnostic accuracy, to interpret children’s performance on tasks designed to assess ICW level and to assist in the selection of more meaningful interventions.

In this paper we focus on children with SLI now referred to in English speaking countries as children with developmental language disorder [22]. In the literature to date the term specific language impairment is used to describe a form of developmental language impairment in which children have difficulty acquiring receptive and or expressive oral language, with or without additional pragmatic difficulties. They may also have pragmatic difficulties. In addition, in order to be given this diagnosis the criteria specify that a child must have adequate hearing and no other specific condition that might interfere with their learning.

Memory research in children with SLI has primarily been carried out with respect to two dominant models, that of Baddeley and Hitch [23–24] and Daneman and Carpenter [25]. Assessment measures used in the current study are based on the Baddeley and Hitch (1974) [24] model.

### Baddeley memory model

In the early Baddeley and Hitch working memory model, working memory is a multidimensional system, composed of three separate but interactive components [26]–the phonological loop, the visuo-spatial sketchpad and the central executive. The phonological loop is responsible for the short-term storage of verbal information. It has a limited capacity and comprises a phonological store and an articulatory rehearsal system. Incoming speech is stored temporarily in the phonological store and is assumed to fade within about 2–3 seconds unless rehearsed by the articulatory rehearsal process. The phonological loop will be referred to henceforth as phonological short-term memory (pSTM). The visuo-spatial sketchpad is responsible for the short-term storage of visuo-spatial information but is not the focus of the current study. The central executive is a resource limited, domain general system and is responsible for the regulation and co-ordination of information in both the phonological loop and the visuo-spatial sketchpad. Together, the functions of the central executive and phonological loop support the temporary storage and processing of verbal information. This is referred to as working memory (WM). Therefore, while pSTM is responsible for the short-term storage of verbal material, WM always involves an additional element of processing. In 2000 Baddeley [27] proposed a new component to the original Baddeley and Hitch model—the episodic buffer. This is composed of a temporary storage system, which is capable of combining phonological and visual information with information from long term memory. The information is then integrated into ‘chunks’, which can be accessed by the central executive. The memory assessment battery used in this study is based on the original Baddeley and Hitch (1974) model [24] and does not include subtests designed to measure this component. For this reason we do not discuss the episodic buffer further.

### Memory in children with LI

Over the past decade or so a memory-based account has been increasingly proposed to explain the sentence comprehension deficits in children with SLI [28–31]. Researchers purporting this view consider comprehension to be an interaction between linguistic abilities and more general cognitive processing and therefore assume an association between sentence comprehension and memory limitations in children with SLI. A pSTM deficit assumes that children with SLI have insufficient storage to retain the contrastive lexical detail of the input resulting in reduced comprehension. Whereas a working memory deficit hypothesis assumes insufficient storage to retain what has been previously processed while continuing to process other linguistic material (see Montgomery et al. [30] for a review) In studies investigating memory in children with SLI specifically in relation to sentence comprehension, results appear mixed. Results from studies carried out by Montgomery [4], Montgomery and Evans [19], Norbury et al. [6], and Robertson & Joanisse [31] suggest a correlation between phonological short-term memory (pSTM) and children’s comprehension of simple (subject-verb-object) constructions while other studies showed no such correlation [16–18]. In relation to more complex structures (such as passives, pronominals and reflexives) Montgomery and Evans [19] reported a significant correlation between working memory and these sentence types. Frizelle and Fletcher [20] reported on memory in relation to relative clause constructions of varying degrees of difficulty and found a significant association between WM and the more complex relative clause constructions and a significant association between pSTM and the least difficult construction. They suggested a synergistic relationship between components of memory and the degree of difficulty of the sentence that is being processed. However, the aforementioned studies are difficult to interpret in relation to the current study as (1) their focus is on children’s understanding of syntactic structures and (2) with the exception of Frizelle and Fletcher [20] they assess both pSTM and working memory using a single measure (rather than a number of measures resulting in a composite score). Considering the memory literature overall, the reported associations between memory and language in children with SLI, and the fact that receptive ICW score is a numeric calculated on the basis of *number of lexical items* the child must act upon in the instruction in order to carry it out correctly, we could reasonably assume an association between children’s ICW score and their memory skills.

However, despite the number of studies that have investigated the relationship between verbal memory and both simple and complex sentences, the issue of how memory might relate to children’s ability to understand sentences containing different numbers of ICWs has never been addressed.

The current paper aims to address the gap in our research and clinical knowledge by attempting to validate ICW as a clinical measure in relation to standardized measures of memory and language.

The following research questions are considered:

1. Is there a relationship between the receptive ICW score of children with SLI and their performance on standardized memory and language assessments?
2. If a relationship does exist what are the relative contributions of language and memory to the ICW scores of children with SLI?
3. Which measure of language and or memory makes the greater contribution to the ICW scores of children with SLI?

## Methodology

### Participants

Forty children with SLI, between the ages of 5;07 and 8;11 years, were recruited in to the study. Thirteen of the children were subsequently excluded as a result of not meeting the SLI diagnostic criteria or failing the hearing screen. The participants included consisted of 17 boys and 10 girls, with a mean age of 7;01 years (SD = 12.57 months). In order to participate in the study children were required to score below -1.25 standard deviations (SD) on the composite scores derived from either the receptive or expressive language subtests of the Clinical Evaluation of Language Fundamentals (CELF-4) [32]. See Table 1 for descriptive statistics.

**Table 1:** Summary of group characteristics and performance scores on standardised sssessments.

Measure	Mean	SD	Min—Max
Age	7;1 years	1.05 years	5;7–8;11 years
IQ SS (measured by Raven’s matrices)	98.89	10.95	85–125
ICW score (out of 240)	193.74	17.05	169–229
Receptive language RS	55.59	11.52	30–71
Receptive language SS	76.74	10.90	57–96
Expressive language RS	68.44	15.33	36–103
Expressive language SS	77.26	7.07	65–93
Phonological short-term memory RS	64.93	10.27	44–78
Phonological short-term memory SS	87.41	12.16	57–110
Working memory RS	25.04	6.36	14–43
Working memory SS	74.30	8.11	55–92

ICW = information carrying word; SS = standard score; RS = raw score.

Of the twenty-seven children, five met the language inclusion criteria based on their receptive language scores, six on expressive language scores and sixteen based on their performance on both receptive and expressive measures. Children were also required to demonstrate cognitive ability within the normal range (achieve a score of 85 or greater on The Raven’s test of Progressive Matrices [33]) and pass a hearing screen in both ears, administered at 25dB and at three frequencies (1000 Hz, 2000 Hz and 4000 Hz). These are the most common frequencies and hearing levels at which young children are consistently screened (as documented by Bamford et al. in the NHS—Current Practice, accuracy and effectiveness report, [34]). Based on speech and language therapy (SLT) reports, children were excluded on the basis of a previous diagnosis of Autistic Spectrum Disorder, Attention Deficit Hyperactivity Disorder, major physical disabilities, an intellectual disability or a hearing impairment. Children were recruited from clinics in a city in Southern Ireland, and were either attending or waitlisted for SLT. Consent to participate was provided in writing by each child’s primary caregiver. Children who participated also completed an assent form. Written ethical approval for the study was obtained from the Cork Teaching Hospitals Clinical Research Ethics Committee.

### Performance measures

Measures relevant to the current study were collected to represent five sets of variables: (1) children’s ability to understand sentences with an increasing number of ICWs, (2) children’s phonological short-term memory skills, (3) children’s working memory skills, (4) children’s receptive language skills and (5) children’s expressive language skills.

#### ICW sentence comprehension task

**Fig 1:**
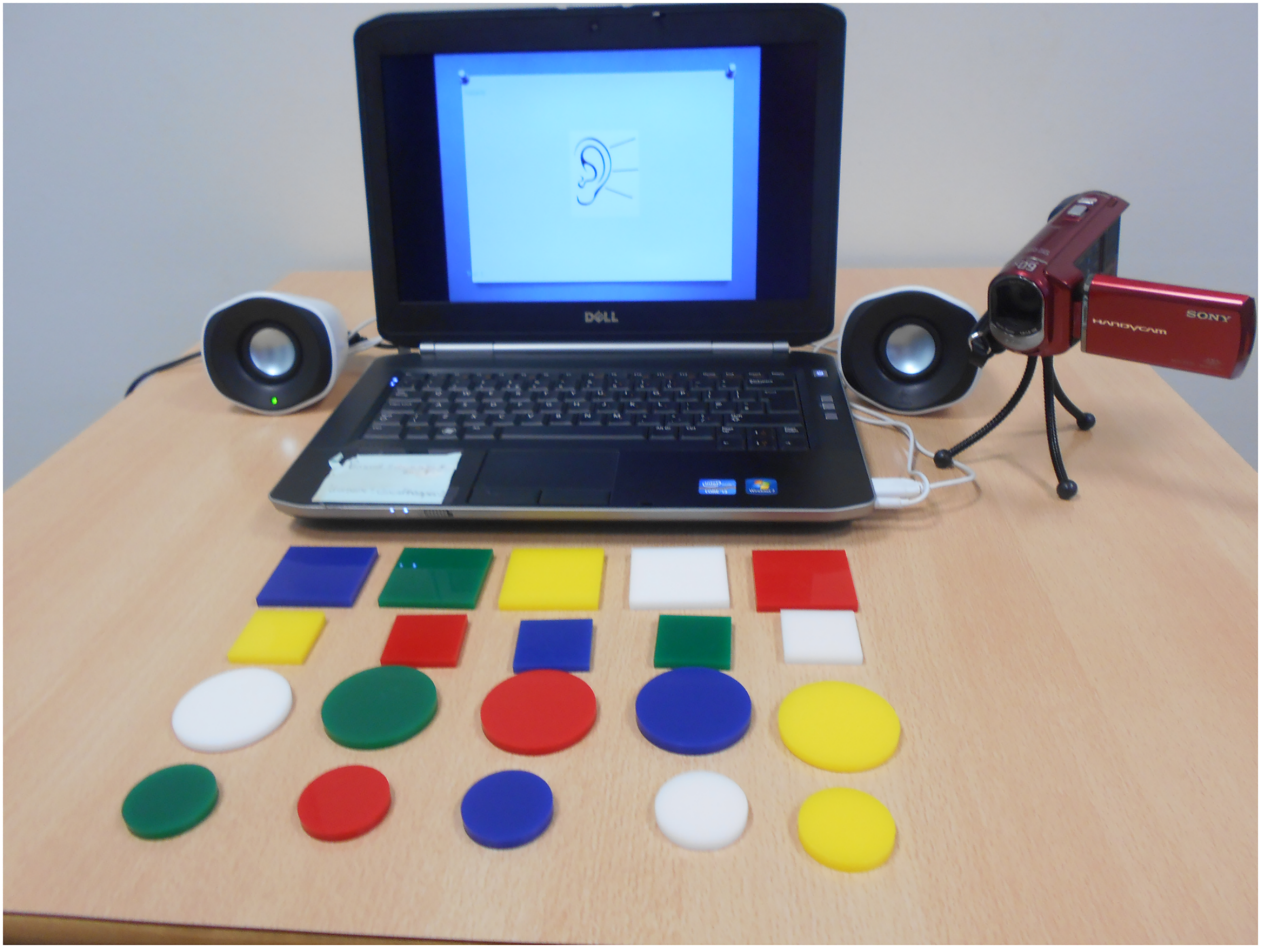
Image of experimental set up and test materials. Set up includes laptop, speakers, video camera and test tokens [35].

The ICW sentence comprehension task was adapted from the Token Test [35] designed for use with children aged 3 to 12 years. Instructions, increasing in the number of ICWs (matched for syllable length), were spoken by two female SLTs of similar age and the children were required to carry out each instruction by manipulating real objects (tokens) of different size, colour and shape. An image of the experimental set up is shown in Fig 1.

The task in the current study consisted of 46 instructions presented in two blocks of 23. An example of the test instructions given is shown in Table 2.

**Table 2:** Examples of test instructions given for ICW task.

Section	Instruction
Section 1	Touch the large blue square
	Touch the small white circle
Section 2	Touch the blue square and the yellow square
	Touch the red square and the white circle
Section 3	Touch the small blue square and the large red square
	Touch the large yellow square and the small red circle
Section 4	After picking up the green square, touch the white circle
	Except for the square to the left of the red square, touch all the squares

Information carrying words (ICWs) are underlined in this table.

The instructions were organised in four sections and increased in the number of ICWs as the child progressed through the task (from three ICWs in section 1 up to 11 ICWs in section 4).

#### Scoring of sentence comprehension task

A score of one was given for each ICW correctly identified within a given instruction. The summation of these scores resulted in a total ICW score for each child. For example given the instruction *Touch the* *large* *white* *square* *and the* *large* *red* *circle* a child could score a total of 6, as there are six ICWs in this sentence (the words *Touch the* are not included as they are used repeatedly throughout the first three sections). However, if (given the same instruction) the child touched the *white* *square* (but not the large one) *and the* *small* *square* (but not the red one) they would score 4. The total score for each child across all sections gave a value on which children could be compared.

#### Memory tasks

Children’s memory functioning was assessed using the Working Memory Test Battery for Children (WMTB-C [36]). This is a standardized memory assessment providing multiple measures of short-term and working memory. The test consists of eight subtests, designed to assess the phonological loop, visuo-spatial sketchpad and central executive components of Baddeley’s [23] model of working memory (for validation study see [37]). The test is not designed to directly assess the episodic buffer. For the purposes of this study the subtests assessing the phonological loop (pSTM) and the central executive (working memory) components were administered.

Phonological short-term memory (pSTM) was measured using four subtests; *digit recall*, *word list matching*, *word list recall* and *non-word list recall*. Three of these measures (*digit recall*, *word list recall* and *non-word list recall*) use an immediate serial recall paradigm where children are asked to temporarily store and then recall digits, words or non-words. A span is then calculated based on the level at which the child can recall. The fourth measure requires that the child judges whether two spoken word sequences are identical or not. All four subtests provide a composite score of pSTM.

Children’s WM skills were measured using the *listening recall*, *counting recall* and *backward digit recall* subtests. All of these measures require both storage and processing of information. The *listening recall* subtest is an adaptation of the Competing Language Processing Task [38]. The child is required to make a truth-value judgement about a sentence presented aurally (for example–*fish can swim*) while at the same time trying to recall the final word in the sentence. The sentences are arranged in groups reflecting six levels of difficulty. In level 1 the child must only understand one sentence and recall the last word of that sentence, while at level six the child must understand all six sentences in the group and then recall the final word of each previously presented sentence. The *counting recall* task (based on that by Case, Kurland and Goldberg [39]) requires the child to count the number of randomly presented target dots in a series of displays and to recall the tally of each presentation. The *backward digit recall* subtest requires the child to repeat a list of digits in reverse order. A span is calculated for each subtest based on the level at which the child can recall. Combined performance on the three subtests is represented in a composite WM score. As we were interested in investigating the memory abilities of children with different ICW scores we did not want to adapt for age, and so we used raw scores rather than standard scores in the statistical analyses.

Although the WMTB-C is a standardized assessment designed to measure pSTM and WM the influence of language on individual subtests has been well documented in the literature. If we consider for example digit span, it is reported that recall of random sequences of numbers is superior to that of sequences of words [40]. This advantage is considered to be a reflection of the increased frequency of random digits in everyday language relative to other verbal material. In addition non-word repetition has long been linked to vocabulary knowledge [41]. Although, research by Melby,—Lervag and colleagues [42] found no causal relationship between NWR ability and vocabulary scores and suggest that it may be a consequence rather than a cause of vocabulary knowledge. The influence of language on NWR repetition ability is further reinforced by Jones [43]. Focussing on linguistic exposure, Jones uses computational modelling to account for changes in NWR performance in children from 2 to 6 years. On the basis of his results he concludes that NWR is a measure of the child’s current level of linguistic knowledge.

Other research showing the phonotactic frequency effects on greater NWR accuracy for word like non-words [44] also suggests that children’s language knowledge affects their ability to accurately do this task. In addition we know that children with impaired language have significant NWR deficits [45–46]. Therefore, although these tests are used throughout the research literature as measures of pSTM, we acknowledge that language ability is an influencing factor in children’s performance on these tasks.

#### Language tasks

The language measures administered were the receptive and expressive subtests of the CELF- 4 [32]. The receptive subtests administered were *Concepts and Following Directions*, *Word Classes—Receptive* and *Sentence Structure*. The *Concepts and Following Directions* subtest assesses a child’s ability to understand spoken instructions of increasing length and complexity, which contain concepts of inclusion / exclusion, location, sequence and time e.g. *Point to the apple in the top row and the fish in the bottom row*. The child is also required to remember the order in which the objects and concepts are given. The *Word Classes—Receptive* subtest examines a child’s ability to understand relationships between words that are semantically related. The child is presented with either three or four named pictures and must identify the two words that go together best e.g. *butterfly*, *caterpillar*, *kitten*. The *Sentence Structure* subtest evaluates a child’s ability to understand spoken sentences increasing in length and syntactic complexity and to select pictures from a choice of four, which represent the meaning of each sentence e.g. *The woman who is holding the baby dropped her handbag*.

The expressive subtests completed were *Word Structure*, *Formulated Sentences and Recalling Sentences*. The *Word Structure* subtest assesses a child’s ability to apply morphological rules, marking inflections, derivations and comparisons e.g. *This man sings*. *He is called a ______*. It also assesses the child’s ability to use appropriate pronouns to refer to people, objects and possessive relationships, e.g. *She is waving at ____ and he is waving at ____*. The *Formulated Sentences* subtest examines the child’s ability to orally generate complete semantically and grammatically correct sentences of increasing complexity, using specific words (e.g. *forgot*, *always*, *when*) and contextually constrained by illustrations given. The *Recalling Sentences* subtest evaluates the child’s ability to listen to spoken sentences of increasing length and complexity and to repeat those sentences verbatim, i.e., without changing the word meanings, morphology or syntax. An example sentence is *The rabbit was not put in the cage by the girl*. Again raw scores were used in the statistical analyses.

In relation to measures of pSTM we have discussed the influence of language knowledge and the difficulty in keeping measures of verbal memory and language separable. As verbal memory is influenced by language, it is also the case that measures of language are influenced by verbal memory. One obvious example of this is the recalling sentences subtest outlined above as part of the CELF-4 test battery. A reliable clinical marker of SLI [12] there is a large body of literature postulating what is involved in the process of recalling sentences. Although ostensibly it appears to be a task involving the reproduction and recalling of a series of heard words, researchers are now converging on the view that it is not the case. Sentence recall is believed to be assisted by lexical, conceptual and syntactic representation in long-term memory [47–52], as well as by phonological short-term memory processes [53–56].

In this study we wanted to validate the ICW construct as a clinical measure in relation to standardized measures of language and memory, which provided multiple measures of each domain and which are used in clinical practice. Based on the Baddeley model the WMTB-C provides scores for both pSTM and WM based on a number of subtests, all of which are designed to have memory as the primary measure. Similarly the CELF- 4 provides a receptive and expressive language score based on a number of subtests but aimed primarily to assess language. We acknowledge that within these standardized measures language and memory are not always entirely separable. However given the broad range of subtests completed in each domain we believe that overall the assessments of both memory and language are accurate reflections of children’s respective abilities in these areas.

## Results

### Descriptive Statistics

All relevant data is available on Open Science Framework https://osf.io/89t2m/

Table 1 provides a summary of the mean, SD and range for each of the measures. Both standard scores and raw scores are given for each of the language and memory measures.

#### Relationships between ICW scores, age, language skills and memory

Table 3 provides a summary of the correlations between the measures.

**Table 3:** Correlations among independent and dependent variables.

Measure	ICW	Age	IQ	Rec	Exp	pSTM	WM
Age	.58**	–					
IQ	.29	-.08	–				
Rec	.73***	.67***	.03	–			
Exp	.68***	.71***	.17	.52**	–		
pSTM	.52**	.603**	.18	.64***	.66***	–	
WM	.72***	.79***	.16	.45*	.602**	.57**	–

* p < .05,

** p < .01,

*** p < .001

ICW = information carrying word; Rec = receptive language; Exp = expressive language; pSTM = phonological short-term memory; WM = working memory.

Correlations between the ICW scores and both the receptive language (r = .73, p < .001) and working memory measures (r = .72, p < .001) were highly significant. Using Davis [57] criteria for interpreting the magnitude of correlation co-efficients these are classified as very strong associations. The correlations between ICW and expressive language scores (r = .68, p < .001) and between ICW and pSTM (pSTM r = .52, p < .01) were also significant and are classified as *substantial associations* [57]. ICW score was also highly correlated with age (again a substantial association). There was no correlation between ICW score and IQ. There was no evidence of collinearity amongst the independent variables. Initial examination of gender indicated that it was not significantly associated with ICW score. Furthermore interactions between gender and each memory and language variable were not significant.

To investigate the independent contributions of age, language skills and memory to ICW scores, a series of hierarchical regression analyses were conducted (regression analyses in which all variables were entered in blocks of related measures) using ICW scores as the dependent variable. In both models age was entered in the first block in order to control for its effect on the children’s performance on the task. In the first model the second and third blocks consisted of receptive and expressive language scores in order to evaluate the role of language skills in ICW score above and beyond the developmental influences of age.

Receptive language was entered first due to its higher correlation with ICW score. The fourth and fifth blocks consisted of the two memory measures; WM and pSTM with a view to assessing the relations between ICW score and memory having accounted for age and overall language ability. Again the higher correlate of the two memory measures (WM) was entered first.

In the second model the order of entry of the language and memory variables was reversed. The second and third blocks consisted of the two memory measures and the third and fourth blocks included the two language measures. As in the first model the higher correlate in each domain was entered into the model first. This allowed us assess the role of memory in ICW score (having accounted for age and language) and evaluate the relationship between ICW score and language over and above the contribution of age and memory. By carrying out the analyses in this manner it allowed us investigate 1) whether memory or language was the greater contributor to children’s ICW score 2) which aspect of each domain contributed more to children’s ICW score. Results of the regression analyses are summarized in Table 4.

**Table 4:** Multiple regression analysis—models 1 and 2.

Model	Variable	R^2^	R^2^ increase	*p*
1	*Block 1*			
	Age	.34	.34	.001
	*Block 2*			
	Receptive language	.54	.21	.003
	*Block 3*			
	Expressive language	.65	.11	.012
	*Block 4*			
	Working memory	.74	.08	.017
	*Block 5*			
	Phonological Short-term memory	.74	.00	.995
2	*Block 1*			
	Age	.34	.34	.001
	*Block 2*			
	Working memory	.52	.18	.006
	*Block 3*			
	Phonological Short-term memory	.54	.02	.388
	*Block 4*			
	Receptive language	.64	.10	.02
	*Block 5*			
	Expressive language	.74	.10	.011

The total model accounted for a large amount of the variance (74%) in the ICW scores and age explained 34% of that variance (*p* < .001). In the first model an additional 32% of the variance was explained by the inclusion of language demonstrating the significant contribution of overall language abilities on ICW score. Receptive language accounted for 21% of this variance (*p* = .003) and expressive language for a further 11% (*p* = .012). The addition of memory accounted for a further 8% of the variance all of which was determined by working memory (*p* = .017). The contribution of pSTM was negligible. A similar memory pattern was revealed in the second model where both memory components were included in blocks two and three in the equation. Following age, WM accounted for a significant 18% of the variance in ICW score (*p* = .006), while pSTM accounted for a mere 2% (*p* = .39). The addition of language accounted for a further 20% of the variance; 10% for each language component and both values were significant—receptive (*p* = .02) and expressive (*p* = .011). In conclusion the analyses showed that in addition to receptive and expressive language, WM also made a significant contribution to children’s ICW scores (having accounted for age). However receptive language was the largest contributor to the variance in ICW score.

Examining the regression co-efficients for the five independent variables (see Table 5), receptive (*p* = .014) and expressive language (*p* = .011) and working memory (*p* = .02) were significant and all three measures were positively associated with ICW. Age and pSTM were not significant.

**Table 5:** Regression coefficients for models 1 and 2.

Variable	β	95% CI	p
Age	-0.57	-1.18–0.05	.069
Receptive language	0.67	0.15–1.19	.014
Expressive language	0.50	0.13–0.87	.011
Phonological short-term memory	-0.002	-0.53–0.53	.995
Working memory	1.29	0.23–2.36	.02

### Discussion

The motivation for conducting this study came from the lack of research into the relationship between ICWs, language and memory skills and therefore an absence of validation in relation to the ICW construct. Addressing this knowledge gap is particularly important due to the recent survey [2] indicating that almost all SLTs in the UK use the ICW construct in the treatment of children’s receptive and or expressive language.

### Relationship between total ICW score, memory and language tests

Our first research question asked whether there is a relationship between the ICW scores of children with SLI and their performance on language and memory tests. A very clear picture emerged. There was a significant relationship between both language and memory measures and children’s ICW score, i.e., the higher the ICW score, the higher the children’s performance on tests of language and memory. The strongest associations were between ICW score and receptive language and ICW score and working memory—both being classified as *very strong associations* [57].

Our second and third research questions asked about the relative contributions of language and memory to the ICW scores of children with SLI. Our results consistently showed that both receptive and expressive language were significant in their contribution to children’s ICW score, however the contribution of memory was solely determined by children’s working memory ability. Therefore we can conclude that based on the standardized language and memory tests used in the current study, ICW score as a measure is closely related to children’s overall language ability (receptive and expressive) and to their ability to remember and process information at the same time using their working memory.

### The Role of language in ICW score

So how do we interpret these findings? Our results suggest that receptive ICW score is in fact a valid measure of the language ability of children with SLI. While therapists may ostensibly assume that this would be the case, it has never been investigated. Previous studies investigating the associations between different language measures report mixed results and it is not always the case that performance on one language measure is a predictor of another [58].

In this study (with the exception of the final section) the set of vocabulary items was primarily limited to colour shape and size. Use of prepositions and verb variability was limited to the final section and the vocabulary items were repeated in different combinations throughout the task. They were also chosen on the basis that they would be familiar to young children and therefore by design were not stretching children to their receptive language limits. In this respect we may not have anticipated an association between children’s ICW scores and their overall language skills, (receptive language skills in particular) but even within this context a strong association emerged.

### The Role of memory in ICW score

Our results suggest that working memory also plays a significant role in children’s ICW scores but that the role of pSTM is negligible. Our analysis shows strong associations for both memory components in the initial correlations. However if we examine the five variables fitted in the regression model we can see the non-significant contribution of pSTM. While other studies have shown correlations between pSTM, WM and sentence comprehension in children with SLI [4, 6, 19], as previously outlined they are difficult to interpret in the context of the current study. Studies researching the relationship between memory and sentence comprehension in children with SLI have tended to focus on syntactic structures, with many assessing each memory component using a single subtest. More importantly, other studies have looked directly at the association between memory and a previously validated measure of language. In contrast, in the current study we were trying to validate ICW score as a measure of language and were therefore interested in the contribution of memory to this construct both when language had and had not been accounted for. Our results consistently show the significant contribution of working memory and the minimal contribution of pSTM both when language had and had not been accounted for. PSTM reflects the child’s ability to temporarily hold linguistic material in mind. We could argue that in the current study its contribution to ICW score may have been diminished due to 1) the restrictive and repetitive nature of the vocabulary utilised in three out of the four sections and 2) the visual supports provided by the tokens, therefore requiring each child to recall the vocabulary from a small lexical set. That is, the task made limited demands on pSTM even in children for whom limitations might have been expected in this area of memory. In relation to working memory the significant contribution to ICW score is perhaps unsurprising. Both the WM and ICW tasks require the child to store an amount of linguistic material in their pSTM and then process that material for meaning by acting on it in some way. Both tasks also require children to use their controlled attention to manage simultaneous storage and processing demands. In addition, measures of working memory such as the listening span task are highly loaded in relation to language. In this task children are required to process the truth-value of blocks of sentences and then to recall as many sentence final words as they can. With each additional sentence the child must update the list of items to be stored. The task requires the ability to switch between processing the sentence for it’s meaning and remembering the appropriate list of items without interference from previous sentences. Similarly the ICW task requires the child to process each instruction for meaning and remember the specific ICWs in the instruction while inhibiting the irrelevant material from previous sentences. Lexical interference has been documented as an influencing factor in the sentence comprehension of children with SLI [29]. In our adapted version of the token test we used a small set of semantically related lexical items repeatedly, which could lead to a considerable amount of interference for children with SLI. This would require the children to engage in the process of inhibition and increase their dependence on working memory.

In summary the concept of contextual redundancy is central to the ICW construct. The strong association between WM and ICW score suggests that even when given instructions specifically controlled for number of ICWs, where other aspects of the sentence are contextually redundant, it is only when they have processed the entire instruction that they can decide which elements of the sentence are contextually accounted for. SLTs should therefore be cognisant of all elements of the sentence when using this construct and not assume the child is immediately aware of contextual redundancy.

## Conclusions and implications

Our findings indicate that ICW score is in fact a valid measure of the language ability of children with SLI. The children in the current study had a mean age of 7,01 years and had cognitive abilities within the average range. It would be useful to investigate if similar associations emerged with a younger group of children and with children functioning below the average range on cognitive measures. It is also noteworthy that while many therapists adapt the ICW construct to their specific therapeutic goals others adhere closely to the intervention programme from which it emerged—The Derbyshire Language Scheme [1]. While some of our instructions mirror those used in the Derbyshire scheme (put the x beside the y) our verb and preposition variability is limited and our sentence types are all command based. It would therefore be interesting to attempt to validate this measure using the specific tasks and instructions from the Derbyshire programme.

Nevertheless our results show that when using the ICW framework SLTs can now be confident that it is associated with other relevant standardized measures of language. ICW score is also strongly influenced by children’s working memory ability and therapists need to be cognisant of this influence when using the ICW construct in either assessment or intervention. It is possible that the association with working memory could be reduced by utilizing a more semantically diverse vocabulary set but this would need further investigation.

## References

[1] Knowles W, Masidlover M. The Derbyshire Language Scheme. Derby: Derbyshire County Council; 1982.

[2] Morgan L, Marshall J, Harding S, Roulstone S. Why do SLTs adapt the therapy they provide? Bulletin. 2013; 738(10): 16–18.

[3] Donlan C, Bishop DVM, Hitch GJ. Magnitude comparisons by children with specific language impairments: Evidence of unimpaired symbolic processing. Int J Lang Commun Disord. 1998; 33(2): 149–160. 9709434 10.1080/136828298247802

[4] Montgomery J. Sentence comprehension in children with specific language impairment: The role of phonological working memory. J Speech Hear Res. 1995; 38: 187–199. 7731209 10.1044/jshr.3801.187

[5] Bishop DVM. Test of Reception of Grammar (TROG). Manchester, England: University of Manchester; 1989.

[6] Norbury C, Bishop D, Briscoe J. Does impaired grammatical comprehension provide evidence of an innate grammar module? Appl Psycholinguistic. 2002; 23: 247–268.

[7] Dunn LM, Dunn DM, Whetton CW, Pintillie D. The British Picture Vocabulary Scales (BPVS). Windsor, UK: NFER Nelson; 1982.

[8] Durkin K, Conti-Ramsden G. Language, social behavior, and the quality of friendships in adolescents with and without a history of specific language impairment. Child Dev. 2007; 78(5): 1441–1457. doi: 10.1111/j.1467-8624.2007.01076.x 17883441 10.1111/j.1467-8624.2007.01076.x

[9] Semel E, Wiig E, Secord W. Clinical Evaluation of Language Fundamentals—Revised (CELF-R). San Antonio, TX: The Psychological Corporation; 1987.

[10] Eisenberg SL, McGovern Fersko T, Lundgren C. The use of MLU for identifying language impairment in preschool children: A review. Am J Speech Lang Pathol. 2001; 10: 323–342.

[11] Riches NG. Sentence repetition in children with specific language impairment: an investigation of underlying mechanisms. Int J Lang Commun Disord. 2012; 47(5): 499–510. doi: 10.1111/j.1460-6984.2012.00158.x 22938061 10.1111/j.1460-6984.2012.00158.x

[12] Conti-Ramsden G, Botting N, Faragher B. Psycholinguistic markers for Specific Language Impairment (SLI). J. Child Psychol. Psychiatry. 2001; 42(6): 741–748. 11583246 10.1111/1469-7610.00770

[13] Leonard L, Ellis Weismer S, Miller C, Francis D, Tomblin J, Kail R. Speed of processing, working memory, and language impairment in children. J Speech Lang Hear Res. 2007; 50: 408–428. doi: 10.1044/1092-4388(2007/029) 17463238 10.1044/1092-4388(2007/029)

[14] Montgomery JW, Windsor J. Examining the language performances of children with and without specific language impairment: Contributions of phonological short term memory and speed of processing. J Speech Lang Hear Res. 2007; 50: 778–797. doi: 10.1044/1092-4388(2007/054) 17538115 10.1044/1092-4388(2007/054)

[15] Gray S. The relationship between phonological memory, receptive vocabulary, and fast mapping in young children with specific language impairment. J Speech Lang Hear Res. 2004; 49: 955–969. 10.1044/1092-4388(2006/069) 17077208

[16] Montgomery J. Relation of working memory to off-line and real-time sentence processing in children with specific language impairment. Appl Psycholinguistic. 2000; 21: 117–148.

[17] Montgomery J. Verbal working memory and sentence comprehension in children with specific language impairment. J Speech Lang Hear Res. 2000; 43: 293–308. 10757685 10.1044/jslhr.4302.293

[18] Montgomery J. Sentence comprehension in children with specific language impairment: Effects of input rate and phonological working memory. Int J Lang Commun Disord. 2004; 39: 115–134. doi: 10.1080/13682820310001616985 14660189 10.1080/13682820310001616985

[19] Montgomery J, Evans J. Complex sentence comprehension and working memory in children with specific language impairment. J Speech Lang Hear Res. 2009; 52(2): 269–288. doi: 10.1044/1092-4388(2008/07-0116) 18723601 10.1044/1092-4388(2008/07-0116) PMC4684953

[20] Frizelle P, Fletcher P. The role of memory in processing relative clauses in children with specific language impairment. Am J Speech Lang Pathol. 2015; 10.1044/2014_AJSLP-13-0153 25409883

[21] Montgomery JW, Polunenko A, Marinellie SA. Role of working memory in children's understanding spoken narrative: A preliminary investigation. Appl Psycholinguistic. 2009; 30(3): 485–509.

[22] Bishop DV, Snowling MJ, Thompson PA, Greenhalgh T, CATALISE consortium. CATALISE: A multinational and multidisciplinary Delphi consensus study. Identifying language impairments in children. PeerJ Preprints. 4: e1986v1 https://doi.org/10.7287/peerj.preprints.1986v1 10.1371/journal.pone.0158753 PMC4938414 27392128

[23] Baddeley A. Working memory and language: An overview. J Commun Disord. 2003; 36: 189–208. 12742667 10.1016/s0021-9924(03)00019-4

[24] Baddeley A, Hitch GJ. Working memory In: Bower GA, editor. Psychology of learning and motivation, volume 8 New York: Academic Press; 1974 pp. 47–89.

[25] Daneman M, Carpenter P. Individual differences in working memory and reading. J Verb Learn Verb Beh. 1980; 19: 450–466.

[26] Bayliss DM, Jarrold C, Baddeley AD, Gunn DM. The relationship between short-term memory and working memory: Complex span made simple? Memory. 2005; 13(3–4): 414–421. 15948627 10.1080/09658210344000332

[27] Baddeley A. The episodic buffer: A new component of working memory? Trends Cogn Sci. 2000; 4(11): 417–423. 11058819 10.1016/s1364-6613(00)01538-2

[28] Epstein B, Hestvik A, Shafer V, Schwartz R. ERPs reveal atypical processing of subject versus object wh-questions in children with specific language impairment. Int J Lang Commun Disord. 2013; 48: 351–365. doi: 10.1111/1460-6984.12009 23889832 10.1111/1460-6984.12009 PMC3728699

[29] Leonard L, Deevy P, Fey M, Bredin-Oja S. Sentence comprehension in specific language impairment: A task designed to distinguish between cognitive capacity and syntactic complexity. J Speech Lang Hear Res. 2013; 56: 577–589. doi: 10.1044/1092-4388(2012/11-0254) 22988286 10.1044/1092-4388(2012/11-0254) PMC3661700

[30] Montgomery JW, Gillam RB, Evans JL. Syntactic versus memory accounts of the sentence comprehension deficits of Specific Language Impairment: Looking back, looking ahead. J Speech Lang Hear Res. 2016; 59(6): 1491–14. doi: 10.1044/2016_JSLHR-L-15-0325 27973643 10.1044/2016_JSLHR-L-15-0325 PMC5399765

[31] Robertson E, Joanisse M. Spoken sentence comprehension in children with dyslexia and language impairment: Roles of syntax and working memory. ‎Appl. Psycholinguist. 2010; 31: 141–165.

[32] Semel E, Wiig EH, Secord WA. Clinical Evaluation of Language Fundamentals– 4, UK Standardisation (CELF–4 UK). London: Pearson Assessment; 2006.

[33] Raven JC. Raven’s—Educational: CPM/CVS. London: Pearson Assessment; 2008.

[34] Bamford J, Fortnum H, Bristow K, Smith J, Vamvakas G, Davies L, et al Current practice, accuracy, effectiveness and cost-effectiveness of the school entry hearing screen. Health Technol Assess. 2007; 11(32): 5–16. 10.3310/hta11320 17683682

[35] Mcghee RL, Ehrler DJ, Disimoni F. Token Test for Children (TTFC– 2). 1st ed Texas: PRO-ED Inc; 2007.

[36] Pickering SJ, Gathercole SE. Working Memory Test Battery for Children (WMTB-C). London: Pearson Assessment; 2001.

[37] Gathercole SE, Pickering SJ, Ambridge B, Wearing H. The structure of working memory from 4 to 15 years of age. Dev. Psychol. 2004; 40: 177–190. doi: 10.1037/0012-1649.40.2.177 14979759 10.1037/0012-1649.40.2.177

[38] Gaulin C, Campbell T. Procedure for assessing verbal working memory in normal school-age children: some preliminary data. Percept Mot Skills. 1994; 79: 55–64. doi: 10.2466/pms.1994.79.1.55 7991333 10.2466/pms.1994.79.1.55

[39] Case R, Kurland MD, Goldberg J. Operational efficiency and the growth of short-term memory span. J Exp Child Psychol. 1982; 33: 386–404.

[40] Jones G, Macken WJ. Questioning short-term memory and its measurement: Why digit span measures long-term associative learning. Cognition. 2015; 144: 1–13. doi: 10.1016/j.cognition.2015.07.009 26209910 10.1016/j.cognition.2015.07.009

[41] Gathercole SE, Willis C, Emslie H, Baddeley A. Phonological memory and vocabulary development during the early school years: A longitudinal study. Dev. Psychol. 1992; 28: 887–898.

[42] Melby-Lervåg M, Lervåg A, Lyster SAH, Klem M, Hagtvet B, Hulme C. Nonword-repetition ability does not appear to be a causal influence on children’s vocabulary development. ‎Psychol. Sci. 2012; 23: 1092–1098. doi: 10.1177/0956797612443833 22923338 10.1177/0956797612443833

[43] Jones G. The influence of children’s exposure to language from two to six years: The case of nonword repetition. Cognition. 2016; 153(C): 79–88. 27155560 10.1016/j.cognition.2016.04.017

[44] Coady J, Evans JL, Kluender KR. Role of phonotactic frequency in nonword repetition by children with specific language impairments. Int J Lang Commun Disord. 2010; 45 (4): 494–509. doi: 10.3109/13682820903222783 19821795 10.3109/13682820903222783 PMC4705560

[45] Bishop DV, North T, Donlan C. Nonword repetition as a behavioural marker for inherited language impairment: Evidence from a twin study. J. Child Psychol. Psychiatry. 1996; 37(4): 391–403. 8735439 10.1111/j.1469-7610.1996.tb01420.x

[46] Conti-Ramsden G. Processing and linguistic markers in young children with specific language impairment (SLI). J Speech Lang Hear Res. 2003; 46(5): 1029–1037. 14575341 10.1044/1092-4388(2003/082)

[47] Brown GDA, Hulme C. Modeling item length effects in memory span: No rehearsal needed? J. Mem. Lang. 1995; 34: 594–621.

[48] Hulme C, Maughan S, Brown GDA. Memory for familiar and unfamiliar words: evidence for a long-term memory contribution to short-term memory span. J. Mem. Lang. 1991; 30(6): 685–701.

[49] Klem M, Melby-Lervåg M, Hagtvet B, Lyster SAH, Gustafsson JE, Hulme C. Sentence repetition is a measure of children’s language skills rather than working memory limitations. Dev. Sci. 2015; 18(1): 146–54. doi: 10.1111/desc.12202 24986395 10.1111/desc.12202 PMC4309482

[50] Potter MC, Lombardi L. Regeneration in the short-term recall of sentences. J. Mem. Lang. 1990; 29: 633–654.

[51] Potter MC, Lombardi L. Syntactic priming in immediate recall of sentences. ‎J. Mem. Lang. 1998; 38: 265–82.

[52] Schweickert R. A multinomial processing tree model for degradation and redintegration in immediate recall. Mem Cognit. 1993; 21: 168–75. 8469125 10.3758/bf03202729

[53] Alloway TP, Gathercole SE. Working memory and short-term sentence recall in young children. Eur J Cogn Psychol. 2005; 17: 207–20.

[54] Hanten G, Martin RC. Contributions of phonological and semantic short-term memory to sentence processing: Evidence from two cases of closed head injury in children. J. Mem. Lang. 2000; 43(2): 335–361.

[55] McCarthy R, Warrington E. The double dissociation of short-term memory for lists and sentences. Brain. 1987; 110(6): 1545–1563. 3427400 10.1093/brain/110.6.1545

[56] Rummer R, Engelkamp J. Phonological information contributes to short-term recall of auditorily presented sentences. J. Mem. Lang. 2001; 45(3): 451–467.

[57] Davis JA. Elementary survey analysis. Englewood Cliffs, NJ: Prentice—Hall; 1971.

[58] Frizelle P, O'Neill C, Bishop DVM. Assessing understanding of relative clauses: A comparison of multiple-choice comprehension versus sentence repetition. J Child Lang. 2017; 1–23 10.1017/S0305000916000635 28088927

